# All the More Reason to Get a Flu Shot: An Instance of Acute Hemorrhagic Leukoencephalitis in a Patient With Influenza A

**DOI:** 10.7759/cureus.12885

**Published:** 2021-01-24

**Authors:** Samantha Kops, Katherine Dunne, Merlin C Lowe

**Affiliations:** 1 Pediatrics, Banner University Medical Center, Tucson, USA; 2 Pediatrics, Banner-Diamond Children's Medical Center, Tucson, USA

**Keywords:** acute hemorrhagic leukoencephalitis, influenza vaccine

## Abstract

Acute hemorrhagic leukoencephalitis (AHLE) is a rare demyelinating disease of the central nervous system that typically follows a viral or bacterial respiratory infection. We report the first described case of AHLE following influenza A (H3N2) in an otherwise healthy 15-year-old girl with no relevant past medical history who initially presented to the emergency department (ED) by emergency medical services (EMS) with decorticate posturing and right gaze deviation after being found unresponsive at home. Subsequent testing for Influenza A H3N2 via viral polymerase chain reaction (PCR) was positive. Clinical correlation and brain MRI confirmed AHLE. At follow-up three months after discharge, she was found to have intellectual functioning in the extremely low range and she still had deficits in motor skills eight months after discharge. While the patient was reportedly up-to-date on her routine scheduled childhood vaccinations, she had not received her annual influenza vaccination that year. Pediatric infectious disease physicians and neurologists should consider the diagnosis of AHLE in unvaccinated, previously healthy patients with new and rapid onset of neurological symptoms following influenza infection.

## Introduction

Acute hemorrhagic leukoencephalitis (AHLE) is an acute, progressive, demyelinating disease of the central nervous system. AHLE was first described by Dr. Hurst in 1941 and is considered to be a hyper-acute form of acute disseminating encephalomyelitis (ADEM). It usually follows upper respiratory infections (such as Influenza) or in rare cases vaccinations [1]. The most common clinical presentation includes headaches, seizures, focal neurological signs, and impaired consciousness several days after viral illness or vaccination [1-3]. Diagnosis is typically made by clinical presentation correlated with positive magnetic resonance imaging (MRI) findings of multifocal acute perivascular demyelination and hemorrhage in cerebral white matter [4]. In cases resulting in death, pathological diagnosis can also be made and shows perivascular demyelination, perivascular ball and ring hemorrhages, fibrinoid replacement of vessel walls, glial nodules, and perivascular neutrophilic leukocyte exudates [3-4]. Cerebrospinal fluid (CSF) can show pleocytosis with elevated protein content [3,5]. Mortality has been found to be up to 50% and death is common within one week of neurological symptoms [4]. Treatment for AHLE includes immediate initiation of intravenous methylprednisolone. Intravenous immunoglobulin (IVIG) and plasmapheresis are often employed as critical adjuncts to steroids [3,6]. Given the acute onset and progressive nature of this disorder, it is important to consider AHLE in previously healthy patients presenting with rapid onset of new neurological symptoms.

## Case presentation

A previously healthy 15-year-old girl with no significant past medical history presented to the emergency department (ED) by emergency medical services (EMS) after a family member found her to be unresponsive in her home. The patient had reportedly suffered a fall in the shower the night prior to the presentation for which she took ibuprofen and went to bed. The following morning, she could not be aroused, and EMS was called. Upon arrival, EMS reported a fixed, rightward deviated gaze, urinary incontinence, and decorticate posturing. The patient was previously healthy until a week prior when she developed a mild cough. Family members denied any other symptoms including fevers, lethargy, or myalgias. Her vaccinations were up to date except for her recommended yearly influenza vaccine. 

**Figure 1:**
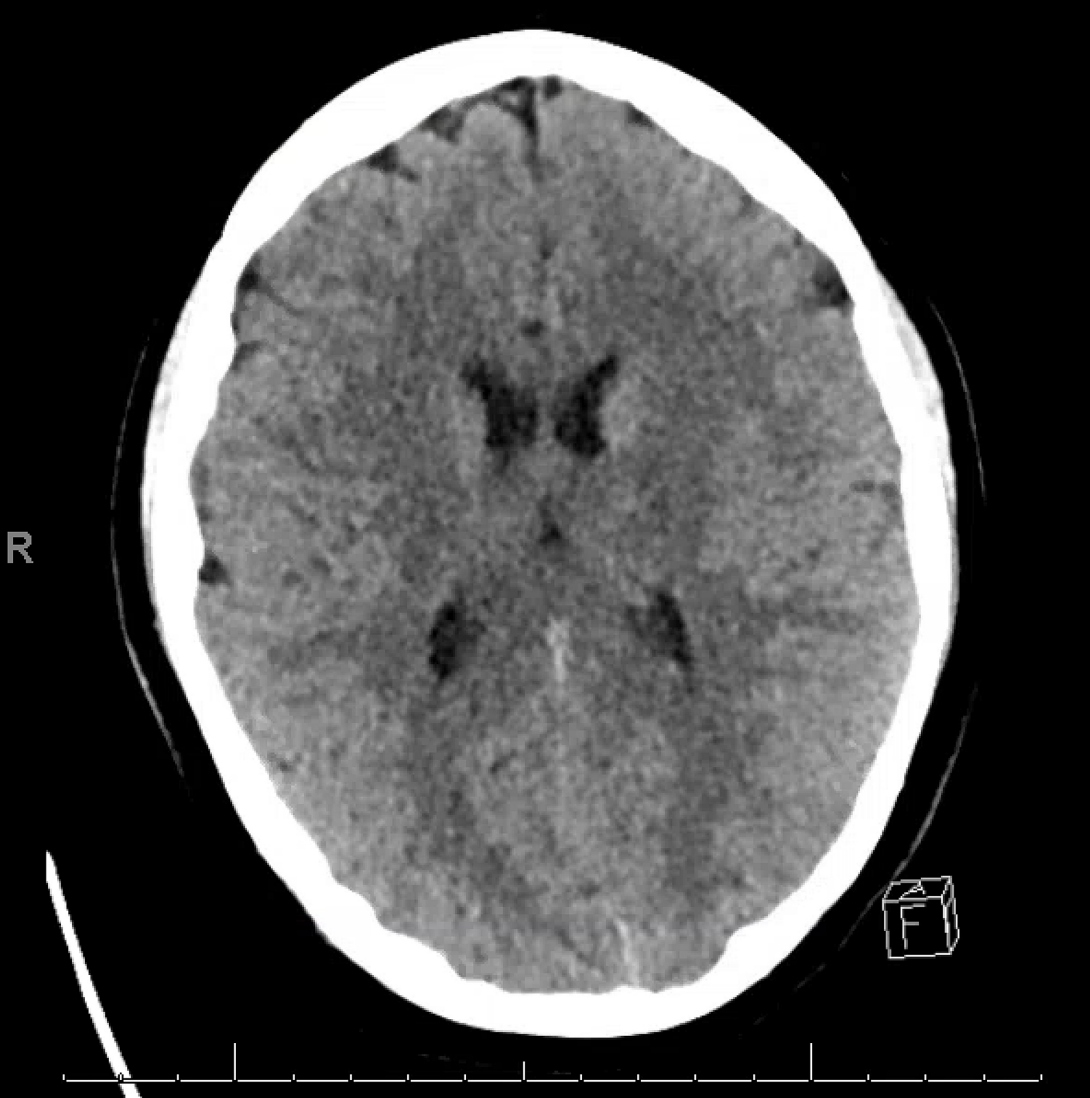
CT of brain at admission with bilateral symmetrical foci of hypodensity within the hypothalamic regions, basal ganglia and thalami bilaterally

On arrival to our facility, the patient was found to have a temperature of 38.3°C, heart rate of 128 beats/min, respiratory rate of 21 breaths/min, blood pressure of 115/51 mmHg, and SpO2 of 99% on oxygen delivered by a non-rebreather face mask at 10 liters per minute flow. Given the presence of decorticate posturing and right gaze deviation, Pediatric Neurology was consulted and 2 mg Ativan and 2 gram Keppra (load) were administered with subsequent mild reduction of gaze deviation and posturing. Cultures were obtained including blood, sputum, urine, and cerebrospinal fluid (CSF). Empiric vancomycin, cefepime, and acyclovir were initiated. CSF was unremarkable with white blood cell count (WBC) of 1/mm3 (0 mm3 - 30 mm3) with differential of 0% neutrophils, 22% monocytes, and 78% lymphocytes, red blood cell count of 1/mm3 (0 mm3 - 10 mm3), glucose of 69 mg/dL (60 mg/dL - 80 mg/dL) and protein of 28 mg/dL (20 mg/dL - 80 mg/dL). Her initial complete blood count showed no evidence of leukocytosis (WBC 7.7 k/mm3 (4.5 k/mm3 - 13.5 k/mm3), normal hemoglobin of 13.5 g/dL (11.5 g/dL - 14.5 g/dL) and platelets of 206 k/mm3 (130 k/mm3 - 450 k/mm3). Complete metabolic panel was normal including normal sodium, potassium, and glucose. A venous blood gas (VBG) was obtained and showed a normal pH of 7.34 (7.35 - 7.45) with a normal CO2 of 41 mmHg (41 mmHg - 51 mmHg). A head computed tomography (CT) was performed which revealed bilateral symmetrical foci of hypodensity within the hypothalamic regions, basal ganglia, and thalami bilaterally (Figure 1). On admission to our pediatric intensive care unit, she was intubated due to ongoing poor mentation and concern for airway protection. 

**Figure 2:**
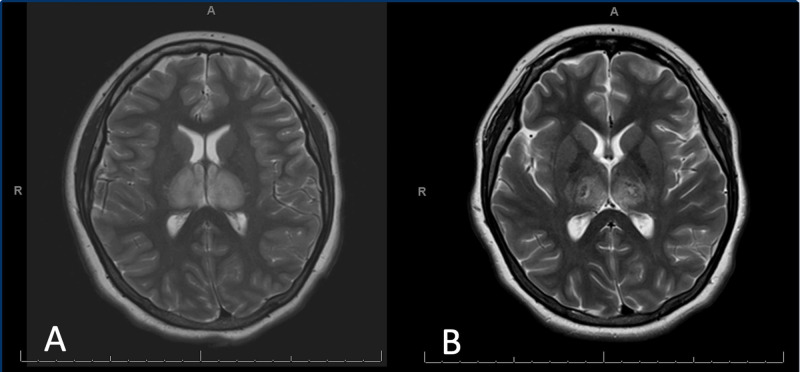
(A) MR brain at admission with central necrosis with hemorrhage in bilateral thalami and hippocampi and restriction with punctate foci of enhancement within pons; (B) MR brain hospital day 10 with evolving necrotic hemorrhagic lesions supratentorially and in the brainstem with no new lesions

Shortly after admission to the pediatric intensive care unit (PICU), magnetic resonance imaging (MRI) demonstrated central necrosis with hemorrhage in the bilateral thalami and hippocampi and restriction with punctate foci of enhancement within pons consistent with AHLE (Figure 2A). Intravenous methylprednisolone was started at a flat dose of 1000 mg daily for a total of five days. Given severe neurological symptoms, the patient was also given one dose of IVIG at 1 g/kg within 24 hours of admission. An infectious disease work-up was initiated and a nasopharyngeal swab was positive for Influenza A (H3N2) via viral polymerase chain reaction (PCR). Acyclovir was discontinued on Day 1 after herpes simplex virus (HSV) CSF PCR was negative. and a five-day course of oseltamivir was initiated. Bacterial cultures of blood, sputum, urine, and CSF were negative at 48 hours so vancomycin and cefepime were discontinued. On day 2 of admission, an EEG demonstrated a background with slow activity which was intermixed with runs of high amplitude delta activity consistent with encephalopathy. IVIG was held while plasmapheresis was continued for a total of five days. After completing the course of plasmapheresis, two more doses of IVIG were given. On hospital day 10 after completing the course of steroids, IVIG, and plasmapheresis, a repeat MRI was performed which showed evolving hemorrhagic necrosis supratentorially and in the brainstem with no new lesions present (Figure 2B).

The patient was extubated on day 13 and transferred to the general pediatric service on day 14. She remained on room air for the rest of her hospitalization. Over the next 12 days, she demonstrated steady improvement in overall neurological status demonstrating partial and intermittent responses to commands such as withdrawing extremities and squeezing fingers when prompted; however, she remained non-verbal and she was unable to eat by mouth. On day 21, the patient underwent surgical G-tube placement to optimize nutrition after discharge. She was discharged to an inpatient rehabilitation facility on Day 26. She completed approximately two months of inpatient rehab and her G-tube was removed during this time as she demonstrated the skills to eat all of her nutrition by mouth. She continued to follow with speech therapy, occupational therapy, and physical therapy and had neuropsychiatric testing approximately three months after discharge which showed overall intellectual functioning in the extremely low range. Her motor skills continued to improve and approximately eight months after discharge, her strength was 4/5 throughout and she was able to take small steps.

## Discussion

AHLE is a demyelinating disease of the nervous system that primarily occurs after a respiratory infection or vaccination and is considered to be an immune-mediated inflammatory condition. It is the most severe form of acute disseminating encephalitis (ADEM) [2,7]. Many viruses including influenza have been associated with ADEM and AHLE in previous reports (specifically H1N1), however, this is the first case described in a patient with Influenza A (H3N2) subtype [1]. 

AHLE is a rare disease and 2% of patients with ADEM have AHLE [5]. The diagnosis of AHLE is more commonly made in young adults and adolescents while patients with ADEM are usually younger children. Both conditions have a male predominance [4,5]. A prodromal illness is more common to ADEM (50%-75% of patients) [8]. Our adolescent patient reported only a mild cough in the days prior to the events leading up to her hospitalization. 

AHLE and ADEM are diagnosed utilizing clinical symptoms and imaging [5]. Clinical characteristics of ADEM include acute encephalopathy with multifocal neurological signs and deficits such as pyramidal signs, hemiplegia, ataxia, cranial nerve palsies, seizures, and altered mental status [3,5,9]. In this case, our patient was nonverbal, unresponsive to painful stimuli, and had pyramidal signs (decorticate posturing) consistent with encephalopathy. ADEM predominately involves white matter of the brain and spinal cord, however, lesions have also been seen in the brain stem, subcortical white matter, thalamus, basal ganglia, and cerebellum (consistent with this case) [3,5,7-8]. MRI in patients with AHLE usually have larger lesions with increased edema, mass effect (26% of patients), and hemorrhages compared to patients with ADEM [3]. Imaging is typically repeated later in the course of the disease and the lesions seen on initial imaging have been shown to resolve in 37%-75% of patients with ADEM after treatment [5]. AHLE was diagnosed in our patient after clinical presentation of severe neurological symptoms and MRI with hemorrhages in the bilateral thalami and hippocampi and restriction with punctate foci of enhancement within the pons (Figure 1). 

Laboratory values, electroencephalograms, and pathology can be a useful tool when diagnosing patients with AHLE. On initial presentation, our patient had a normal CSF distribution (seen in about 24% of patients with ADEM); however, literature has shown CSF in ADEM and AHLE can have pleocytosis (lymphocytic more common in ADEM and neutrophilic more common in AHLE) and elevated protein in some cases [3,5,7-8]. Our patient had diffuse slow background activity found on EEG which has been found in about 65% of patients with ADEM [7,9]. Although our patient did not have a biopsy of her brain performed, pathology can also be potentially diagnostic in patients with AHLE. In ADEM, the infiltrations seen on pathology are usually lymphocyte-predominant while in AHLE they are usually neutrophil predominant [3]. AHLE in particular has also been shown to have perivascular ring and ball hemorrhages, focal areas of demyelination, and fibrinoid replacement of vessel walls and glial nodules [3].

Treatment for AHLE and ADEM consists of immediate high-dose corticosteroids. Rapid treatment is associated with a recovery in ADEM patients, 50%-80% of whom had complete resolution [3,5,8,10]. IVIG and plasmapheresis (as performed in our patient) have also been shown to be efficacious when patients do not respond completely to steroids [5-6,10]. Aggressive and early treatment is important in patients with AHLE as patients can have rapidly progressive brain edema after encephalopathic symptoms and there is increased mortality (up to 50%) compared to patients with ADEM [3-5]. In surviving ADEM patients, the average recovery period is 1-6 months with a 25%-33% risk of relapse [8]. Our patient received rapidly initiated steroids, IVIG and plasmapheresis, and experienced an improved neurologic outcome.

## Conclusions

In conclusion, AHLE can be a devastating disease with an acute onset and progressive nature. Pediatricians, including pediatric infectious disease physicians, emergency medicine physicians, and neurologists should consider AHLE in previously healthy patients presenting with first-time neurological symptoms. Prompt recognition and treatment of AHLE is necessary due to high mortality rates in patients with the disease. This case demonstrates the risk of significant neurological damage that can result secondary to influenza and the possible preventative benefit of an annual influenza vaccine in reducing the risk of developing AHLE following influenza infection is yet to be determined.
